# Harmonization and standardization for selection of high-trueness estradiol assay kits in serum and evaluation of estradiol levels relative to follicle diameters on trigger days

**DOI:** 10.3389/fmed.2026.1867311

**Published:** 2026-07-13

**Authors:** Mengxi Guo, Zhengquan Wang, Xin Chang, Xinxin Ren, Manman Zhang, Yu Tao, Hong Pan, Ningling Wang, Ying Guo, Haixia Ding, Yu Xiao, Yiling Ke, Dandan Wu, Xiaojun Chen, Li Wang, Qing Zhang, Qinhua Zhang, Wen Li

**Affiliations:** 1 Reproductive Medical Centre, The International Peace Maternal and Child Health Hospital, Shanghai Jiao Tong University School of Medicine, Shanghai, China; 2 Shanghai Key Laboratory of Embryo Original Diseases, Shanghai, China; 3 Shanghai Standardization and Harmonization Center of In Vitro Diagnostic, Shanghai, China; 4 Shuguang Hospital, Shanghai University of Traditional Chinese Medicine, Shanghai, China

**Keywords:** 17β-estradiol, Bland-Altman plot-based harmonization algorithm, follicle diameter, harmonization, trueness

## Abstract

**Background:**

The trueness of serum estradiol (E2) measurement is critical for monitoring follicle development in assisted reproductive technology (ART). This study aimed to evaluate E2 assay standardization, assess the trueness of harmonized E2 results across platforms, and investigate the relationship between estimated E2 levels and follicle diameters on human chorionic gonadotropin (hCG) trigger days.

**Methods:**

Serum samples from 90 individuals were analyzed using assays from four manufacturers and LC-MS, which served as the reference method. A Bland-Altman plot-based harmonization algorithm (BA-BHA) was applied to harmonize E2 results. Trueness was assessed by mean percent difference and 95% limits of agreement (LoA). Harmonized results were compared to identify high-trueness kits. Additionally, 237 serum samples with corresponding follicle data from ART patients on hCG trigger days were analyzed. Multiple linear regression was applied to establish the relationship between harmonized E2 levels and follicle diameters.

**Results:**

Before harmonization, mean percent differences from LC-MS ranged from−2.3% to 17.4%. LiCA-E2 demonstrated the best performance. Following harmonization using the BA-BHA, LiCA maintained superior trueness, as indicated by a mean percent difference of 0.1% and a sum of 95% LoA of 41.4%. Multiple linear regression revealed a positive correlation between estimated E2 levels and follicle diameters. LiCA exhibited the strongest linear correlation between estimated E2 levels and corresponding diameters of follicle, with a linear regression equation and a Pearson's correlation coefficient r = 0.8077.

**Conclusions:**

Harmonization effectively improves E2 assay comparability. LiCA's superior performance and strong E2-follicle correlation offer a reliable tool for predicting follicle maturation, guiding clinical decisions in ART.

## Introduction

17β-estradiol (E2) is one of the most biologically active estrogens, primarily secreted by the granulosa cells of ovarian follicles with a peaking level just prior to ovulation (1, 2). Assisted Reproductive Technology (ART) relies heavily on the measurement of serum E2 levels at various critical stages, including ovarian function assessment, follicle development monitoring, timing of egg retrieval, and endometrial status evaluation prior to transplantation (3, 4). Follicle diameters are indicative of follicular development (5). Optimal timing for triggering ovulation is typically determined when there are three mature follicles (≥17 mm in diameter) or two mature follicles (≥18 mm in diameter) (6). Typically, the E2 level in a mature follicle ranges between 734 to 1,468 pmol/L, though this can vary based on individual differences (7).

Beyond its role as a biomarker of follicular growth, serum E2 concentration is an important determinant of several key clinical decisions during controlled ovarian stimulation (COS). In routine ART practice, serial E2 measurements are interpreted together with ultrasonographic follicular monitoring to determine the optimal timing for human chorionic gonadotropin (hCG) triggering. Inadequate or premature triggering may compromise oocyte maturation, whereas delayed triggering may increase the risk of post-mature oocytes and adversely affect treatment outcomes. Therefore, accurate estimation of E2 production from developing follicles is clinically relevant for optimizing oocyte yield and maturity.

In addition, serum E2 levels are widely used for assessing the risk of ovarian hyperstimulation syndrome (OHSS), one of the most serious complications associated with ART. Excessively elevated E2 concentrations, particularly when accompanied by a large cohort of growing follicles, are commonly regarded as indicators of increased OHSS risk and may prompt clinicians to modify treatment strategies, including cycle cancellation, coasting, gonadotropin-releasing hormone (GnRH) agonist triggering, or freeze-all approaches. Consequently, substantial fluctuations or inaccuracies in E2 measurements may influence risk stratification and clinical decision-making.

Furthermore, E2 dynamics provide important information for individualizing ovarian stimulation protocols. Variations in E2 response may reflect differences in ovarian sensitivity to gonadotropins and are frequently considered when adjusting gonadotropin dosage during stimulation. Reliable assessment of the relationship between follicular development and E2 secretion could therefore improve individualized treatment strategies and enhance both efficacy and safety in ART cycles. It is generally understood that larger follicles secrete more E2 (8–10). However, reports on the relationship between the estimated E2 level and the corresponding follicle diameter are still inconsistent, which is urgently needed to be investigated.

For serum E2, there are several reference materials (RMs) from Japan and Belgium and higher-order reference methods including various types of mass spectrometry (MS), such as isotope dilution (ID) gas chromatography (GC)-MS, ID liquid chromatography (LC)-MS, LC-MS/MS, and ID LC-MS/MS, which approved by the Joint Committee for Traceability in Laboratory Medicine (JCTLM) (11, 12). However, methodological factors, differences in measurand, and nonspecific interferences often result in significant discrepancies among the results yielded by different assays (13–16). As a result, harmonization is necessary for E2 measurements.

Recently, a novel protocol, known as the Bland-Altman plot-based harmonization algorithm (BA-BHA), has been proposed for immunoassay harmonization using patient samples as reference materials (17). In this study, we applied BA-BHA to achieve harmonized E2 results among different manufacturers and evaluated the trueness. Furthermore, we investigated the relationship between estimated E2 levels and corresponding follicle diameters on human chorionic gonadotropin (hCG) trigger days, aiming to provide valuable references for clinical decision-making and the evaluation of treatment effects in ART.

## Materials and methods

### Apparatus and reagents

The E2 assay was run on LiCA^®^ 800 (Reagent Lot No. 2304), Beckman DxI 800 (Reagent Lot No. 366248), Roche Cobas^®^ e601 (Reagent Lot No. 10-76718303) and Siemens Atellica^®^ IM (Reagent Lot No. 08056099).

### Ethical approval

Sample and data collection were approved by the ethics committee of the International Peace Maternal and Children Hospital (GKLW-A-2023-053-01).

### Sample preparation

A total of 327 human serum samples, together with corresponding follicular information (including follicle size and number assessed by ultrasonography) and relevant clinical data, were collected from patients undergoing ART on the day of hCG triggering between January and June 2024 at the Department of Assisted Reproduction, International Peace Maternity and Child Health Hospital. Patients underwent ovarian stimulation using either an antagonist protocol or a progestin-primed ovarian stimulation (PPOS) protocol. Patients receiving medications known to affect estrogen levels (e.g., letrozole, oral estrogen formulations such as estradiol valerate, or transdermal estradiol gel) were excluded. Baseline patient characteristics are summarized in Supplemental Table 1.

**Table 1:** Current situation of the standardization of E2 assays.

Manufacturer	Bland Altman plot		Passing-Bablok regression	
	Mean	95% LoA, %	Slope (95% C.I.)	Intercept (95% C.I.)
LiCA	−1.4	−23.7 to 20.9	0.984 (0.950 to 1.028)	−2.306 (−37.820 to 30.160)
Beckman	−2.3	−52.7 to 48.2	1.073 (1.024 to 1.109)	−109.477 (−252.622 to −9.738)
Roche	8.4	−36.7 to 53.6	1.176 (1.121 to 1.231)	−124.284 (−309.857 to −18.065)
Siemens	17.4	−28.9 to 63.7	1.282 (1.252 to 1.310)	−139.959 (−219.203 to −67.882)

From these samples, 90 (designated as Group A) with concentrations ranged from 25.18 to 34509.03 pmol/L evenly distributed across the entire measurement interval (IQR, 985.37–9641.40) were selected as harmonization reference materials (HRMs). These HRMs were used to harmonize E2 results and evaluate the trueness (the closeness of agreement between the mean of measured values and the true value) of harmonized E2 results in serum across various manufacturers. The remaining 237 samples (designated as Group B) were utilized to investigate the relationship between estimated E2 levels and corresponding follicle diameters on hCG trigger days.

Serum was separated from blood cells within 2 h of collection and aliquoted into 0.5 ml tubes without pooling or filtration. All samples were promptly frozen at −80 °C after aliquoting. Each aliquot underwent only one freeze-thaw cycle. Samples exhibiting obvious hemolysis, icterus, or chylemia were excluded from the study.

### Value assignment of HRMs by LC-MS

The 90 samples in Group A, designated as the HRMs, were measured using LC-MS at the National Center for Clinical Laboratory (NCCL). As described in previous studies (14), the LC-MS system was calibrated using certified reference material NMIJ CRM 6004-a (Assigned Value 0.984 kg/kg, Expanded Uncertainty 0.003 kg/kg), which is provided by the National Metrology Institute of Japan according to the JCTLM. The internal standard solution used was 17β-[16, 16, 17-d3] estradiol (isotope abundance > 99%), purchased from CDN Isotopes (Québec, Canada). The resultant LC-MS values were assigned to the HRMs.

### Current situation of the standardization of E2 assays

The 90 HRMs in Group A were subsequently measured using four kits indicated above, following manufacturers' instructions. The E2 results from each manufacturer were compared with the assigned HRM values using the Bland-Altman (B-A) plot and Passing-Bablok regression analysis. Considerations in the evaluation of test equivalence included following factors:

– The mean difference between the manufacturer's results and the assigned HRM values was close to zero in the B-A plot.
– For the sum of the 95% LoA in the B-A plot, the smaller it was, the more equivalency the test.
– “0” was included in the 95% confidence interval (C.I.) of the intercept and “1” was included in the 95% C.I. of the slope in the Passing-Bablok regression analysis.
– Scatter points in the B-A plot were symmetrically and evenly distributed on both sides of the mean line.

### Harmonization of E2 results and evaluation of trueness

The BA-BHA protocol was applied to harmonize E2 results, as described in previous studies (17). Following harmonization, E2 results from each manufacturer were compared with assigned HRM values using the B-A plot to evaluate trueness based on CLSI EP09c-Ed3 (18, 19). Trueness was evaluated according to the requirements of above criteria.

### Investigation of the relationship between E2 levels and follicle diameters

The remaining 237 serum samples in Group B were measured using kits from four different manufacturers after harmonization. Multiple linear regression was applied to estimate E2 levels of different follicle diameters, using harmonized E2 results as the dependent variable and the quantity of follicles of different diameters as multiple independent variables. The regression coefficient of each independent variable was considered as the estimated E2 level of the follicle with the corresponding diameter. Simple linear regression was then used to determine the relationship between the estimated E2 levels and corresponding follicle diameters.

### Statistical analysis

The current situation of standardization and the trueness of harmonized E2 results were evaluated using the B-A plot and the Passing-Bablok regression. The relationship between the estimated E2 levels and corresponding follicle diameters was investigated using multiple linear regression and simple linear regression.

All hypothesis testing was two-tailed and *P* value < 0.05 was considered to be statistically significant. B-A plot and Passing-Bablok regression were conducted using MedCalc^®^, version 20.022 (MedCalc Software Ltd.). Multiple linear regression and simple linear regression were conducted using SPSS^®^ Statistics, version 19 (IBM Corp.). Histograms were plotted using Origin Pro^®^, version 2021 (Origin Lab Corp.).

## Results

### Current situation of the standardization of E2 assays

**Figure 1:**
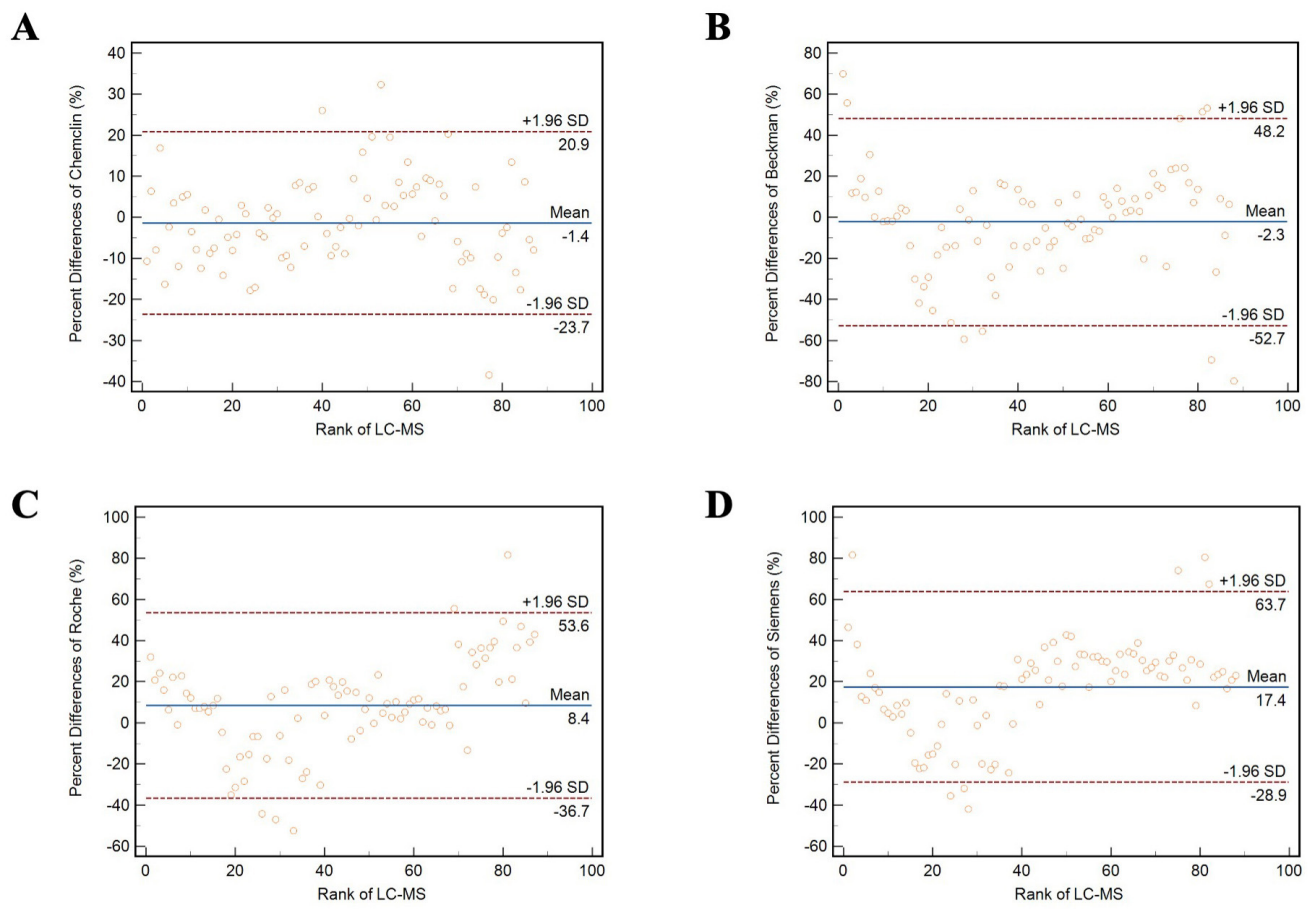
The Bland-Altman plots of E2 results under the condition of current standardization. Data were plotted by paired-comparisons between LS-MS measuring values and assay results from each candidate manufacturer before harmonization. **(A)** LiCA; **(B)** Beckman; **(C)** Roche; **(D)** Siemens.

As shown in Table 1, the mean difference and sum of 95% LoA between measured results from each manufacturer and LS-MS were−1.4% and 44.6% for LiCA, −2.3% and 100.9% for Beckman, 8.4% and 90.3% for Roche, and 17.4% and 92.6% for Siemens, respectively. The regression analysis showed that LiCA presented a slope around the targeting value of “1” and an intercept close to “0”, while Beckman, Roche and Siemens yielded slopes and intercepts deviating more from the targets. Additionally, the scatter points in the B-A plots illustrated significantly deviated distributions over the measurement intervals for Beckman, Roche and Siemens (Figure 1).

### Harmonization of E2 results and evaluation of trueness

After harmonized by BA-BHA, equivalent E2 results with LC-MS were obtained for all of the four manufacturers (Table 2). The trueness performance of LiCA was the best with the mean difference of 0.1% and sum of the 95% LoA of 41.4%, compared to 0.1% and 89.8% for Beckman, −0.4% and 56.7% for Roche and 0.1% and 52.4% for Siemens, respectively.

**Table 2:** Harmonization of E2 results.

Manufacturer	Bland Altman plot		Passing-Bablok regression	
	Mean	95% LoA, %	Slope (95% C.I.)	Intercept (95% C.I.)
LiCA	0.1	−20.6 to 20.8	0.988 (0.969 to 1.015)	4.756 (−21.760 to 24.290)
Beckman	0.1	−44.8 to 45.0	1.049 (1.010 to 1.084)	−21.152 (−132.222 to 2.332)
Roche	−0.4	−28.7 to 28.0	1.008 (0.985 to 1.031)	1.447 (−24.514 to 16.560)
Siemens	0.1	−26.1 to 26.3	0.978 (0.956 to 0.998)	11.101 (−5.753 to 54.811)

**Figure 2:**
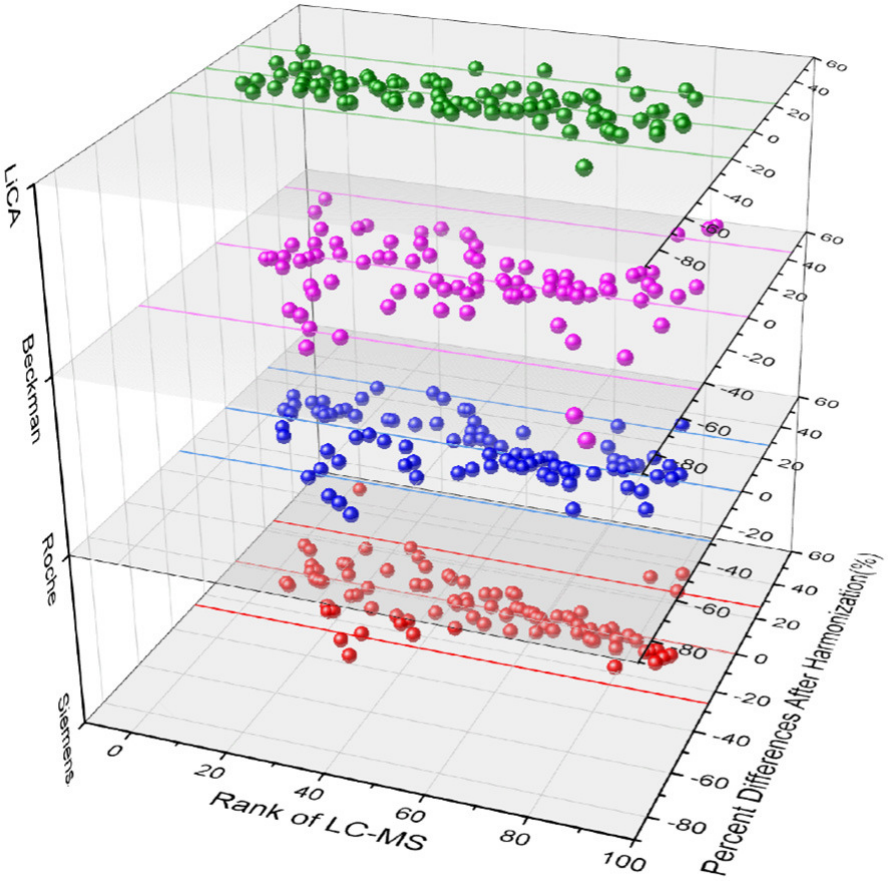
The Bland-Altman plots of E2 results after harmonization. Data were plotted by paired-comparisons between LS-MS measuring values and assay results from each candidate manufacturer after harmonization.

For LiCA, scatter points in the B-A plot were symmetrically and evenly distributed on both sides of the mean line (Figure 2). Additionally, the Passing-Bablok regression indicated an excellent harmonization effect, as “0” was included in the 95% C.I. of the regression intercept and “1” was included in the 95% C.I. of the slope.

### Investigation of the relationship between E2 levels and follicle diameters

**Figure 3:**
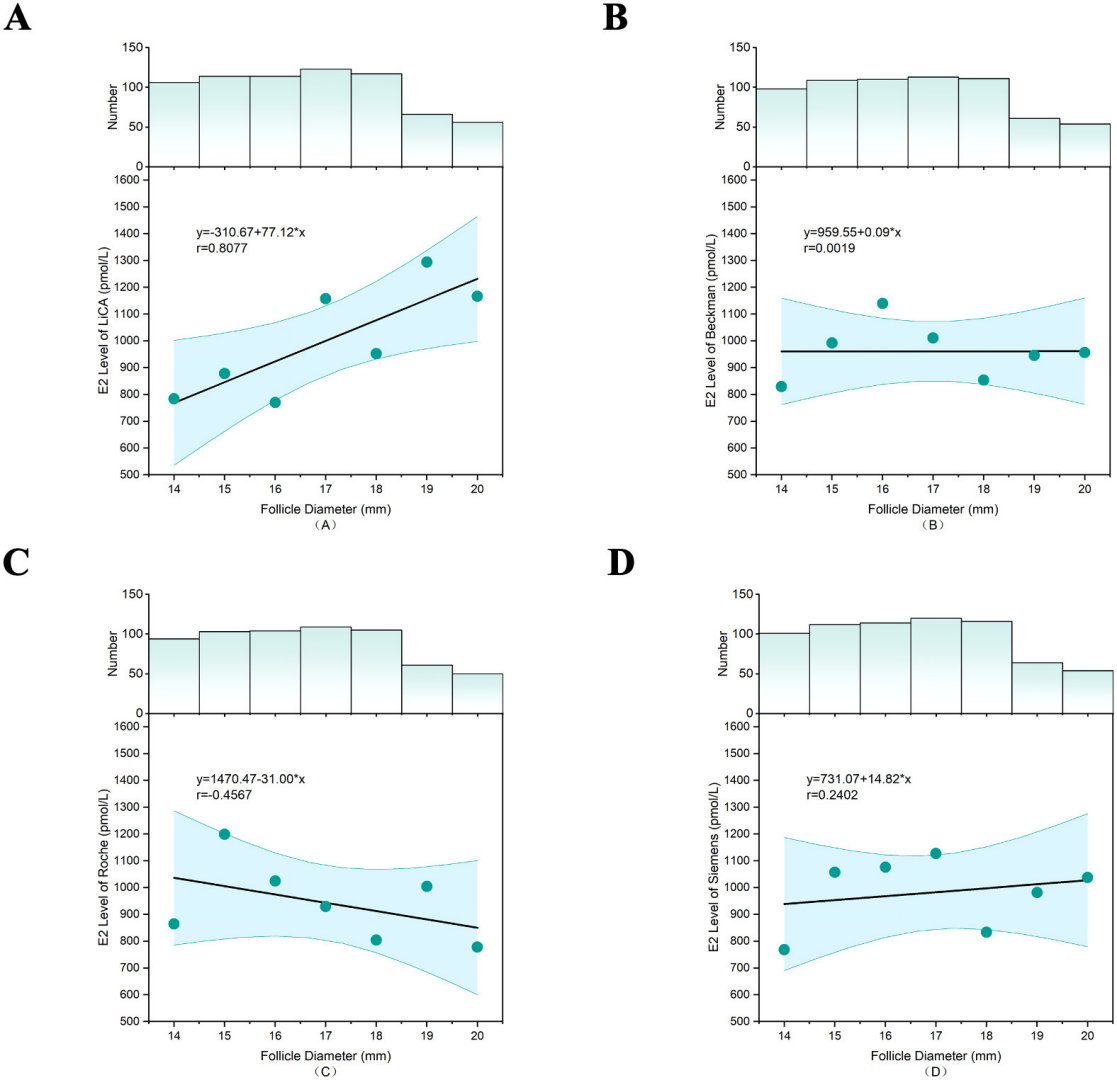
Relationship between the estimated E2 levels and the corresponding diameters of follicles. Multiple linear regression was applied to estimate the E2 levels upon different follicle diameters, using the harmonized E2 results as the dependent variable and the quantity of follicles of different diameters as multiple independent variables. Simple linear regression was then used to determine the relationship between the estimated E2 levels and the corresponding follicle diameters. **(A)** LiCA; **(B)** Beckman; **(C)** Roche; **(D)** Siemens.

Results of linear regression was presented in Table 3. Serum E2 levels were estimated upon different follicle diameters from 14 mm to 20 mm. Then, the relationship between estimated E2 levels (y, pmol/L) and follicle diameters (x, in a range of 14-20 mm) was described with a simple linear regression equation. As shown in Figure 3, LiCA demonstrated the strongest linear correlation with a regression equation y = −310.67+77.12x and a Spearman's correlation coefficient r = 0.8077. In comparison, the linear regression results were y = 959.55+0.09x (r = 0.0019) for Beckman, y = 1,470.47–31.00x (r = −0.4567) for Roche, and y = 731.07+14.82x (r = 0.2402) for Siemens, respectively.

**Table 3:** Results of multiple linear regression for estimated E2 levels in serum upon different follicle sizes.

Follicle diameter, mm	LiCA			Beckman			Roche			Siemens		
	*n*	Estimated E2 level (95% C.I.), pmol/L	Standard error	*n*	Estimated E2 level (95% C.I.), pmol/L	Standard error	*n*	Estimated E2 level (95% C.I.), pmol/L	Standard error	*n*	Estimated E2 level (95%C.I.), pmol/L	Standard error
14	106	784.0 (479.5–1,088.6)	154.2	98	829.4 (47.3–1,111.6)	142.8	94	864.1 (502.0–1,226.1)	183.2	101	768.0 (478.1–1,057.9)	146.8
15	114	878.5 (517.6–1,239.3)	182.7	109	992.0 (646.3–1,337.7)	174.9	103	1,199.3 (789.1–1,609.5)	207.6	112	1,057.5 (722.7–1,392.4)	169.6
16	114	770.2 (449.0–1,091.5)	162.6	110	1,139.2 (844.1–1,434.3)	149.4	104	1,024.6 (636.4–1,412.7)	196.4	114	1,075.8 (770.0–1,381.7)	154.9
17	123	1,157.4 (856.1–1,458.8)	152.6	113	1,011.1 (725.1–1,297.1)	144.7	929	929.8 (572.2–1,287.4)	181.0	120	1,126.9 (843.5–1,410.3)	143.5
18	117	952.2 (614.2–1,290.2)	171.1	111	853.7 (531.5–1,176.0)	163.1	105	803.9 (407.9–1,200.0)	200.5	116	833.2 (523.7–1,142.8)	156.7
19	66	1,293.8 (872.7–1,715.0)	213.2	61	946.0 (526.6–1,365.4)	212.3	61	1,004.4 (506.7–1,502.1)	251.9	64	981.5 (573.7–1,389.2)	206.5
20	56	1,166.2 (633.4–1,699.0)	269.7	54	956.1 (456.7–1,455.5)	252.8	50	778.2 (185.5–1,370.9)	299.9	54	1,037.9 (526.4–1,549.4)	259.0

## Discussion

E2 is instrumental in several physiological processes, including maturation of the female reproductive system, maintenance of secondary sexual characteristics, regulation of reproductive system functions, and promotion of follicular growth and development (9). In the bloodstream, the majority of E2 is bound to sex hormone-binding globulin or albumin, with only a small fraction existing as free, biologically active E2. This free fraction is responsible for interacting with target cells and receptors to exert its physiological effects (20). Measurement of serum E2 plays an essential role in clinical applications, including disease diagnosis, treatment, and risk assessment. Due to its small molecular weight, E2 has full traceability to the International System of Units (SI), allowing for the standardization of E2 assays in human samples. Despite the long-standing use of LC-MS as reference methods for E2, significant differences persist in results among different measuring devices from various manufacturers, which indicates that equivalent assays for E2 have not yet been achieved. It has been reported that around 57% (*n* = 14) calibrators of E2 from four studied manufacturers significantly deviated from the corresponding assigned values, with deviations of 50% (*n* = 14) exceeding 20% (21). Our study also demonstrated varying degrees of percent differences among the results of four E2 assays from those obtained via LC-MS. These differences make medical decision levels assay-dependent, leading to the more complicated triage for the interpretation of test results.

Harmonization, as a special case of standardization, aims to achieve equivalent measured values. It has been proposed by ISO 21151:2020 to follow the guideline of the weighted Deming-based harmonization algorithm (WD-BHA). The new protocol BA-BHA, however, has been suggested in previous studies using as a viable alternative to optimize harmonization for immunoassays (17, 22). Herein BA-BHA was applied to achieve the harmonization among four E2 assays using LC-MS results as the reference. LiCA-E2 demonstrated the highest trueness, with the smallest mean percent differences and sum of 95% LoA after harmonization. The high trueness of E2 assays has important clinical value, particularly for the correct risk prediction of ovarian hyperstimulation syndrome (23–25). Light-initiated chemiluminescent assay (LiCA) is a homogeneous immunoassay that incorporates various cutting-edge technological advances, including nanoparticle polymers, light-initiated chemiluminescent signaling technology, and the unique wash-free immunoassay technique. As a new generation of homogeneous, wash-free chemiluminescent analytical technology, the LiCA avoids errors caused by the washing process during the antigen-antibody reaction (11, 26). Additionally, the LiCA-E2 assay is developed following the similar methodology as LiCA-β-hCG, which integrates reagent antibodies with different affinities, to enable a broadening measurement interval (27). And the metrological traceability of LiCA-E2 has been established in accordance with ISO 17511:2020 (16, 28). This enables accurate detection of the measurand with various concentrations.

During a follicle growth cycle, the diameter of a follicle reflects its development and the E2 concentration in serum is a crucial indicator for monitoring follicle maturation. Serum E2 levels also play a significant role in deciding the appropriate time for triggering (29). It has been reported that the serum E2 level increases by approximately 30.5 pmol/L as the follicle diameter increases by every millimeter (30). By contrast, another retrospective cross-sectional study has illustrated that, in patients undergoing natural cycle *in vitro* fertilization (NC-IVF), the E2 level increases by 167.0 ± 11.0 pmol/L as the follicle diameter increases by 1.04 ± 0.03 mm (8). Our findings may explain the inconsistent relationship between the serum E2 concentration and the corresponding follicle diameter reported. Using different manufacturers' assays such as Beckman, Roche and Siemens, measured E2 values can be significantly deviated from the reference of LS-MS with mean differences from−2.3% to 17.4% and sum of LoAs up to 90.3%−100.9% before harmonization. Furthermore, the poor correlation (r = 0.0019, −0.4567 and 0.2402) between the E2 levels and follicle sizes may not be powerful to estimate a consistent relation for these two factors.

In our study, we established a multiple linear regression model to explore the relationship between follicle diameters and E2 concentrations on the day of hCG administration using the LiCA due to its excellent trueness against LS-MS. Results confirmed a clear linearity between serum E2 concentrations and follicle diameters (r = 0.8077). This demonstrates that a strong correlation between E2 measured results and follicle diameters can be achieved with the advances in methodology and technology. Maybe in the future, we can use the function relation together with numbers of follicles of different diameters obtained from B ultrasound to predict E2 level and then establish a basic AI model to predict the maturity of follicles by comparing the predicted E2 level with the corresponding measured E2 value.

In conclusion, test equivalency of serum E2 appears not applicable although there are several higher-order RMs and reference methods available. Harmonization serves as an essential and effective complement to the standardization of E2 assays. This study offers a valuable reference for the widespread application of a combined approach for immunoassay standardization and harmonization in clinical settings. Compared to Beckman, Roche and Siemens, LiCA presents superior trueness for serum E2 assays using LS-MS as the reference. The robust linear correlation between LiCA measurements and follicle diameters makes it possible for easier prediction of the follicle maturation, providing critical insights for informed clinical decision-making and the evaluation of treatment efficacy in ART.

## Data Availability

The datasets presented in this article are not readily available because & of privacy and ethical restrictions. Requests to access the datasets should be directed to Wen Li, liwen@shsmu.edu.cn.
